# Gapped alignment of protein sequence motifs through Monte Carlo optimization of a hidden Markov model

**DOI:** 10.1186/1471-2105-5-157

**Published:** 2004-10-25

**Authors:** Andrew F Neuwald, Jun S Liu

**Affiliations:** 1 Cold Spring Harbor Laboratory, 1 Bungtown Road, P.O. Box 100, Cold Spring Harbor, NY 11724, USA; 2 Department of Statistics, Harvard University, 1 Oxford Street, Cambridge MA, 02138, USA

## Abstract

**Background:**

Certain protein families are highly conserved across distantly related organisms and belong to large and functionally diverse superfamilies. The patterns of conservation present in these protein sequences presumably are due to selective constraints maintaining important but unknown structural mechanisms with some constraints specific to each family and others shared by a larger subset or by the entire superfamily. To exploit these patterns as a source of functional information, we recently devised a statistically based approach called contrast hierarchical alignment and interaction network (CHAIN) analysis, which infers the strengths of various categories of selective constraints from co-conserved patterns in a multiple alignment. The power of this approach strongly depends on the quality of the multiple alignments, which thus motivated development of theoretical concepts and strategies to improve alignment of conserved motifs within large sets of distantly related sequences.

**Results:**

Here we describe a hidden Markov model (HMM), an algebraic system, and Markov chain Monte Carlo (MCMC) sampling strategies for alignment of multiple sequence motifs. The MCMC sampling strategies are useful both for alignment optimization and for adjusting position specific background amino acid frequencies for alignment uncertainties. Associated statistical formulations provide an objective measure of alignment quality as well as automatic gap penalty optimization. Improved alignments obtained in this way are compared with PSI-BLAST based alignments within the context of CHAIN analysis of three protein families: G_i*α *_subunits, prolyl oligopeptidases, and transitional endoplasmic reticulum (p97) AAA+ ATPases.

**Conclusion:**

While not entirely replacing PSI-BLAST based alignments, which likewise may be optimized for CHAIN analysis using this approach, these motif-based methods often more accurately align very distantly related sequences and thus can provide a better measure of selective constraints. In some instances, these new approaches also provide a better understanding of family-specific constraints, as we illustrate for p97 ATPases. Programs implementing these procedures and supplementary information are available from the authors.

## Background

As the genome projects continue to generate sequence data, it is increasingly common to find protein superfamilies with thousands of members in the protein database. Given sufficient numbers of sequences, sensitive iterative search and alignment procedures, such as PSI-BLAST [1] and SAM [2], often reveal that protein families previously thought to be distinct are, in fact, distantly related. Protein structural analysis likewise reveals subtle evolutionary relationships between protein families sharing very little sequence similarity. Since our ability to make protein structure and function predictions depends in large part on alignment accuracy, it is thus important to develop alignment methods able to handle these increasingly large and diverse sets of distantly related sequences.

Certain protein families within these large superfamilies are often very highly conserved across distantly related organisms. Such proteins include, for example, certain metabolic enzymes, DNA replication and repair factors, certain structural proteins, such as actin, the motor protein dynein, and regulatory and signalling factors, such as protein kinases and Ras-like GTPases. While many of these proteins seem relatively well characterized, we still cannot account for the strong selective constraints preserving their observed high degree of sequence conservation across major taxonomic groups. Presumably these patterns of conservation contain implicit information regarding still unknown functional mechanisms. To access this information, we recently developed a statistically based approach, called contrast hierarchical alignment and interaction network (CHAIN) analysis [3], that identifies, categorizes, and statistically characterizes co-conserved patterns in multiple alignments. The power of this approach strongly depends on the quality of the alignment, which thus motivated the development of the theoretical concepts and strategies described here.

Aligning distantly related sequences presents unique algorithmic and statistical challenges because such proteins often only share a minimal structural core with sizable insertions occurring between, and even within, core elements. Classical dynamic programming-based multiple alignment procedures typically have considerable difficulty spanning across these insert regions because the log-odds scores associated with weakly conserved core elements are often too low to offset the substantial gap penalties that such insert regions incur. This problem is further exacerbated when core elements contain short insertions or deletions within them.

To address this problems, we previously devised motif (or block) based multiple alignment procedures [4-6] that can easily jump over non-homologous insert regions. This approach seems easier to justify than attempting to align regions for which there is no statistical evidence of relatedness. A block based alignment strategy thus seeks to detect islands of subtle sequence similarity within otherwise dissimilar sequences. Fortunately, even when the conserved motifs are very subtle, such a procedure can take advantage of large numbers of available sequences to detect weak, yet statistically significant similarities.

Altschul at the National Center for Biotechnology Information (NCBI) likewise sought to address this problem through generalized affine gap costs [7], but the utility of this approach is unclear, as the NCBI currently does not support any public programs based upon it. The programs MUSCLE [8,9] and MAFFT [10] also are designed to avoid alignment of non-homologous regions and in other respects are generally superior to more widely used multiple alignment programs, such as Clustalw [11] and T-coffee [12]. Because MUSCLE and MAFFT can handle large data sets, we explored the use of these programs for CHAIN analysis (Neuwald, unpublished). Somewhat surprisingly, these failed to achieve the degree of accuracy needed to detect subtle, co-conserved patterns, such as those recently identified and structurally confirmed within P loop GTPases [3]. We found that, although these programs align regions globally conserved in the sequences well, for several large test sets they fail to accurately align regions conserved only within more closely related subsets. This is, of course, a major drawback to their general application for CHAIN analysis. By contrast, PSI-BLAST [1], which seems less likely to produce high quality global alignments given its simple alignment procedure nevertheless in many cases does a better job of aligning database sequences relative to the query. Thus PSI-BLAST (albeit with some modifications to improve alignment accuracy [3]) has turned out to be more generally useful than these other methods for CHAIN analysis, which like PSI-BLAST is query centric. Note, however, that a systematic comparison of various methods within the context of CHAIN analysis has not yet been done.

More relevant to our purpose here, another drawback to the use of MUSCLE, MAFFT, and similar programs for CHAIN analysis is that these will align randomly generated sequences – a characteristic incompatible with the statistical basis of CHAIN analysis. MUSCLE and MAFFT perform well on small sets of relatively diverse representative sequences, such as the BALIBASE benchmark sets [13], because they incorporate heuristics that unfortunately also can compromise statistical rigor and, as a result, confuse random noise with biologically valid homology. Statistically the best alignment for random sequences is the 'null alignment', that is the procedure should leave such sequences unaligned – a property of PSI-BLAST that played a key role in choosing it for CHAIN analysis.

To maintain statistical rigor in our formulations here, we will 'let the data speak' by modelling only those characteristics of the sequences that can be justified by the input data. Such an approach cannot be applied, however, to small benchmark alignment sets, because these lack sufficient sequences – less than the number of amino acids whose parameters are being estimated. Thus, while a rigorous statistical approach has severe limitations when applied to small datasets, it works very well when applied to large, diverse sets of distantly related sequences, as demonstrated, for example, by some of our earlier analyses [14-16].

Two other theoretical issues, which are important to the multiple alignment problem, are devising an objective measure of alignment quality and an efficient strategy for finding the best alignments based on this measure. Our previous methods [4-6] addressed these issues using a Bayesian statistical approach for modelling an arbitrary number of multiply aligned ungapped blocks, each of arbitrary length, in conjunction with a Gibbs sampling procedure for exploring the 'space' of all such alignments. Gibbs sampling is a Markov chain Monte Carlo (MCMC) method that iteratively realigns the sequences with probability proportional to how much the model is thereby improved. Theoretically, beginning from an arbitrary starting alignment, this process will ultimately sample alignments according to the posterior distribution defined by our Bayesian model. Exploring the alignment space in this way is more efficient than taking a greedy approach (one that always chooses the transition to the best alignment) because an element of chance allows the sampler to maneuver around locally optimal traps.

Within this MCMC sampler we implemented specific operations on the alignments, including those allowing for realignment of a sequence against the alignment model, shortening or lengthening of blocks, and creation of recombinant alignments. Such operations function like catalysts to help the sampler avoid or more quickly escape from local optima. Here we expand on the number of these operations and modify our Bayesian model to allow for short insertions or deletions within blocks. In theory, such an approach could be used to sample representative multiple alignments from the posterior distribution, which is relevant to CHAIN analysis because this could be used to adjust position-specific amino acid frequencies for alignment uncertainty. Doing so for the model and operations described here, however, is non-trivial and thus is a topic for a future publication built upon this one. Our primary objective here is merely to obtain the optimal alignment. Thus we also introduce various annealing-like strategies for luring the sampler toward optimum alignments. These include simulated annealing, which is applied within sampling routines, and other intervention strategies. Our primary motivation for developing and implementing these concepts and strategies is to improve CHAIN analysis, as is illustrated here for G-protein *α *subunits, which belong to the P loop GTPase class [17], prolyl endopeptidases, which belong to the *α*,*β*-hydrolase fold class [18,19], and transitional endoplasmic reticulum (p97) ATPases [20], which belongs to the AAA family [21-23] within the AAA+ class [14,24,25].

### Problem definition

The fundamental problem addressed here is to identify the essential features – the common structural core – characteristic of a large set of distantly related proteins. Given an input sequence set, we build a Bayesian statistical model with adjustable parameters to reflect the relationships among the proteins. We also design a stochastic search algorithm, with an MCMC sampler as its backbone, to explore possible alignments and corresponding model parameters in order to find alignment models that best 'explain' the input data. The model parameters specify, for example, the number and lengths of the motifs, their locations within each sequence, the residue frequencies observed at each position in each motif, and other properties (described below). We may thus envision our sampler as searching through a discrete space where each point, corresponding to a particular alignment, has a probability associated with it. The probability function appears fairly smooth inasmuch as nearby points (similar alignments) have roughly comparable probabilities. As the sampler traverses from one point to another, it favors moves toward the better alignments, that is, toward that part of the alignment space with greater posterior probability. Since it is computationally prohibitive for the sampler to consider many transitions at one time, a key design issue is the selection of allowed transitions between points.

## Results and discussion

### The block-motif model

We first define the alignment model in precise mathematical terms, which provides a scoring scheme that allows us to judge which alignment is better than another. Here, for the sake of conciseness and readability, we will keep the discussion on a conceptual level whenever possible. Interested readers can consult our earlier publications for further details [4,6].

**Figure 1:**
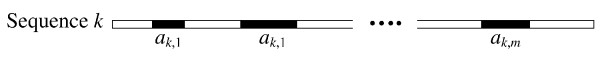
The structure of a sequence containing *m *ungapped motifs denoted *a*_*k*,1 _to *a*_*k*,*m*_.

As illustrated in Fig. 1, this previously described block-based motif model assumes that the aligned core of each protein sequence consists of *m *co-linear ungapped motifs, of width *w*_1_,... , *w*_*m*_, respectively. Each motif is modelled by a position specific frequency matrix Θ_*i*_, whereas residues outside the motif blocks follow a common frequency distribution. Independent prior Dirichelet distributions are employed for these frequency parameters.

Since both *m *and the *w*_*i*_'s are unknown, we assume that they are uniformly distributed in a certain range *a priori *(see [6] for details). We also employ a "fragmentation model," which allows non-informative aligned columns to be ignored by the motif model. Although we use no explicit gap penalties between motifs, our prior imposes a large penalty on alignments with large *m*. Let **S **denote the sequence data and let **A **denote the motif alignments (which also includes *m *and the *w*_*i*_'s). Then the posterior alignment distribution is:

*P*(**A **| **S**) ∝ *P*(**A**) ∫ *P*(**S **| **A**, Θ)*P*(Θ)*d*Θ.

Based on this distribution, our algorithm (as implemented in the PROBE program [5]) attempts to maximize *P*(**A **| **S**), the so-called "*maximum a posteriori *(MAP)" score.

### Hidden Markov models for gapped motifs

**Figure 2:**
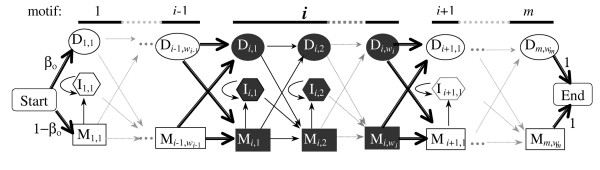
General architecture for our multiple motif HMM. States and transition probabilities between states are defined in Methods. Bold transition arrows emit residue strings.

A major drawback of the previous block-based alignment approach is that it disallows insertions or deletions within motif blocks. Here we describe hidden Markov model (HMM) [26,27] structures for insertions and deletions, which will be used by our current algorithm via the operation GAPALIGN (see below). The general architecture for these HMMs is given in Fig. 2, and detailed descriptions, including the definition for our scoring function *g*(**A**, Λ), are given in Methods.

For an intuitive notion of how within-motif penalties influence the total MAP score, consider a gap-opening penalty of say 20 bits (i.e., *p *= 1/2^20^) and an extension penalty of 2.5 bits. Then, for example, the overall MAP would need to improve by 25 bits in order to justify a 'surgical operation' on a sequence involving an insertion of three dummy residue (i.e., to 'correct' a deletion in a sequence) or a deletion of three residues (i.e., to 'correct' an insertion in a sequence). The statistical problem is thus that of finding the right penalty so that the sampler only adds insertions or deletions when the data provides sufficient justification. In a Bayesian context, this justification is based on the posterior inference of the overall number of insertions and deletions from what it finds in the aligned sequences.

### Markov chain Monte Carlo methods

The Bayesian analysis described in Methods provides us the posterior distribution of the alignment up to a normalizing constant. Although this distribution defines the answer to our problem, namely inferring the optimal alignment, it is difficult to make sense out of it because of the huge size of the alignment space. Fortunately, recent progress in using MCMC methods for statistical analysis has made it possible to study this function.

MCMC methods, of which the Gibbs sampler is a special case, refer to a set of techniques developed by physicists since the 1950s to simulate variables from a given probability distribution up to a normalizing constant. The central idea of these techniques is to evolve a Markov chain, each step of which perturbs the current state (alignment) slightly, with the equilibrium distribution of the chain being the target distribution. A MCMC scheme is usually constructed in two steps: (i) propose a new state according to a certain reversible transition rule, and (ii) accept or reject the proposal according to the probability ratio between the proposed and the current states [28].

The broad utility and general applicability of these techniques are exemplified and popularized by recent developments in statistics: if one can sample from *g*(**A**, Λ) one obtains a set of "typical" alignments according to the posterior distribution, which provides information regarding the most likely alignment(s) supported by the data and its variability. In practice, however, one may wish to find the optimum of this function and explore only around this optimum considering the difficulty of summarizing a set of distinct alignments in a meaningful way. MCMC is also an important ingredient of an optimization technique termed "simulated annealing" [29], of which we will develop a variation. A good MCMC scheme should have the following property: (a) its transition rules should collectively allow the sampler to access every point in the space; (b) these transitions should also allow for global changes, such as, for example, recombination between two alignments; and (c) the acceptance rate of these proposals should be reasonable (10~50%). The sections below will focus on designing such transitions for multiple alignment.

### An algebraic system for touring the alignment space

The elementary mathematical operations of addition and subtraction define a means of transitioning between points in the discrete space of natural numbers. "Global" operations, such as multiplication and integer division, allow transitions between more distant points in this space. Likewise, we define both elementary and global operations on multiple alignments as a means of transitioning between points in alignment space. In this case a set of unaligned sequences (termed the null alignment) serves the same role as the natural number zero. Formal mathematical descriptions of the alignment and of certain simple operations are provided in our earlier papers [4,6]. Since the new operations described here involve various combinations of these simple operations, it is straightforward to derive these new operations from the previously published descriptions.

There are two issues to consider in the design of multiple alignment operations. First, the reversibility of MCMC algorithms requires that every operation have an "inverse" so that the sampler can readily transit in either direction. Second, to help find the optimal alignment according to our Bayesian model, which is our main objective, annealing techniques and less restrictive acceptance rules should be considered for certain complex operations. By doing so the target alignment distribution has to be distorted to some degree, though the global optimum of the distribution remains the same.

All alignments described here are collinear multiple alignments (CMAs), which are defined to contain zero or more motif blocks arranged collinearly in each sequence. Partial or complete deletion of any motif from a particular sequence is modelled by aligning that motif against null residues ('-'), which the sampler may insert anywhere in the sequence. Sequences may also contain more than one repeat of the entire protein domain, each of which is modelled by the full set of motifs. (The identification of repeat domains will be described elsewhere; Spouge and Neuwald, unpublished.) For clarity, we describe operations deterministically, though it should be kept in mind that our sampler applies these stochastically.

#### Elementary operations

The HideInsert operation (inverse ShowInsert) is applied to 'surgically' remove a region of the sequence that appears to correspond to a typically short insertion within a conserved motif. This operation thus changes the real sequence into an idealized sequence that, presumably, more closely resembles the canonical characteristics of the protein class. As a result, the sampler needs to maintain both a real and an idealized version of each protein's sequence and to store the operational derivation used to obtain the ideal sequence from the real. Algorithmically it is convenient to deal with insert regions in this way because otherwise the sampler would need to look up the locations of insertions and deletion within each sequence when applying other operations. The FillDeletion operation (inverse UnfillDeletion) likewise converts a sequence that contains a deletion of either part of or all of a motif into an idealized sequence in which the deletion has been filled in with null or 'dummy' ('-') residues. Note that HideInsert and FillDeletion merely define data structure interconversions that allow basic operations, which were initially defined for ungapped motifs, to be efficiently applied to gapped motifs.

The Align operation assigns motif positions within a sequence and thereby adds that sequence to the alignment, UnAlign removes the sequence from the alignment. Note that these operations *disallow *gaps within motif blocks.

The AddColumn and DeleteColumn operations add and remove aligned columns, respectively. Note that these operations may add or remove columns internal to a motif as well as at the edges. Moreover, AddColumn may also insert a column an arbitrary number of residues beyond the current edge of a motif. This is important for motif 'fragmentation' [4,30], a procedure that allows certain nonconserved positions inside of a motif to be ignored by the alignment statistical model.

#### Compound operations

Elementary operations can be combined in a coordinated manner in various ways to produce compound operations that better facilitate escape from local traps. For example, GapAlign (inverse UngapAlign) combines the row operation Align with the sequence operations HideInsert and FillDeletion in order to add a sequence to an alignment with insertions and deletions. The GapAlign operation is performed using dynamic programming to obtain a gapped alignment of a sequence against a statistical model of the current alignment. The trace back procedure determines how to apply the HideInsert and FillDeletion operations to the true sequence and how the Align operation is then applied to the resultant idealized sequence.

We define several compound operations on a motif block: AddBlock, ShiftRight, and TrimRight (with inverses: DeleteBlock, ShiftLeft, and TrimLeft, respectively). Another compound operation, MoveColumn, which transfers a column from one position to another within a block, is its own inverse. Conceptually, AddBlock and DeleteBlock simply iteratively apply the AddColumn and DeleteColumn operations, respectively. Because our motif alignments are collinear, the position of an added block within each idealized sequence must be specified in a manner consistent with this collinear arrangement and, in order to add a new block in this way, the sampler may need to insert null residues at certain positions within some of the idealized sequences. This is an example of operational flexibility. Similar operational flexibility is required for the ShiftRight and ShiftLeft operations, which remove one or more columns from one end and append it to the other end of a motif. TrimRight and TrimLeft allow poorly conserved residues to be trimmed from a motif block based on their relative entropy. These operations thus provide a means to manually edit motif-based alignments as discussed below.

**Figure 3:**
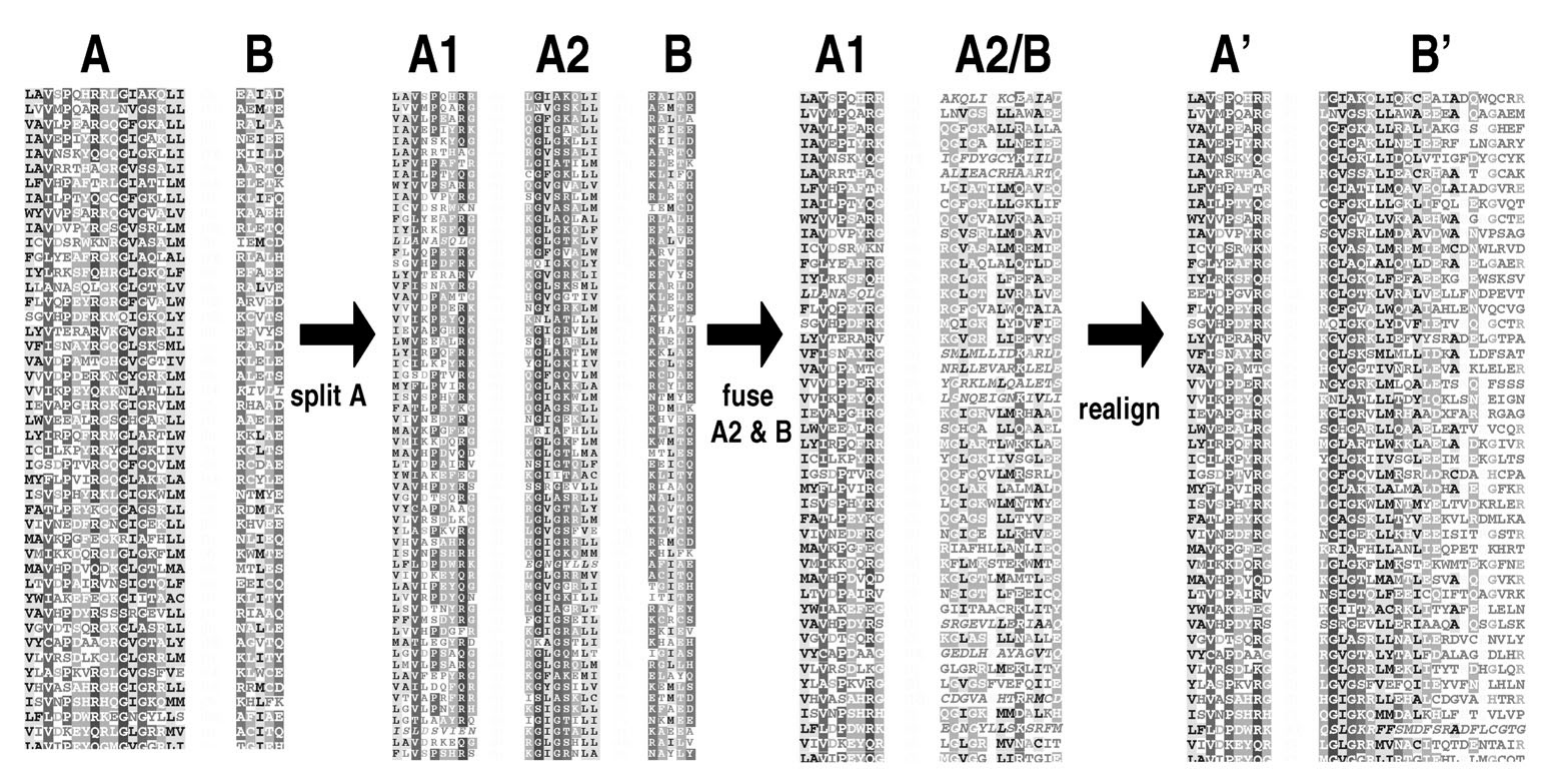
An example of a series of compound operations applied to a motif based alignment. This series splits and fuses blocks in order to escape from kinetic traps in alignment space. See text for details.

Three compound operations involving two motif blocks are: TransferColumn, Splitblock and FuseBlocks. TransferColumn deletes a column from one block and adds it to another block. Splitblock splits a single block into two leaving two contiguous motif blocks in each of the idealized sequences. During future realignment operations the sampler typically induces these abutted blocks to drift apart. Splitblock's inverse operation, FuseBlocks, merges two blocks into one, which typically requires forced realignment of motif positions in each sequence in order to join the blocks together. All such forced realignments are followed by additional optimization via sampling prior to deciding whether to reject or accept this new configuration. We thus typically have to violate the MCMC's acceptance-rejection rule to enable such a move, which distorts the target distribution. The awkwardness of this procedure may be advantageous, however, inasmuch as it forces the sampler out of local traps in alignment space. Fig. 3 illustrates the effect of applying compound operations during Gibbs sampling.

#### Recombinational operations

As an aid to locating the optimum alignment, we define recombination operations that combine the best features of two distinct, fairly well refined alignments. These operations require that the sampler first generate a population of fairly well refined alignments starting from distinct, randomly selected points in alignment space. All of these input alignments must, of course, contain the same set of sequences.

The Recombine operation must be applied to two alignments that are fairly similar because the sampler needs to locate at least one crossover point between them. A crossover point is a set of positions, one position in each aligned sequence, such that the same set of blocks in the first alignment lie to the left of each of those points, while the same set of blocks in the second alignment lie to the right of each point. Because this requirement often proves difficult to satisfy for every sequence, we define the Recombine operation flexibly by allowing a certain number of sequences to violate this rule. In this case, violating sequences are removed prior to recombination and sampled back in afterwards (using the GAPALIGN operation).

The Intersect operation takes as input two distinct alignments and produces a new alignment containing only those aligned columns common to corresponding motifs in *both *input alignments. More precisely, we first find the common blocks shared by the two alignments, where a common block is defined as two aligned motif blocks (one in each alignment) that overlap within corresponding sequences. To allow for some flexibility, these are defined as blocks for which at least some minimum fraction (say 50%) of the sequences are consistently aligned in both input alignments. (Inconsistently aligned sequences are removed from the alignment prior to performing this operation.) Then, for each pair of common blocks, we find the sub-block shared by both blocks. Next, we create a new alignment containing only these Intersecting sub-blocks. Finally, sequences that were inconsistently aligned between the two starting alignments are sampled back into the resulting alignment. The Intersect operation allows the sampler to be reinitialized starting with a consensus alignment that aligns only those regions with high likelihood scores and eliminates those regions about which the sampler is less certain. Subsequent sampling will then extend these sub-blocks, add new blocks, and explore more extensively the alignment space.

#### Parameter settings for operations

There are no absolute rules on how to choose parameter settings for these algebraic operations, such as, for example, the maximum increase in motif length allowed during the MoveColumn operation or the number of disordered blocks to tolerate for the Recombine operation. We find, in fact, that it often matters little which settings are used and the slight degree to which it does matter depends on the particular protein class being analyzed. As a result, any biologically reasonable parameter settings work well. For example, since weakly conserved motifs are never a hundred residues long, motif blocks typically should be limited to no more than, say, fifty residues in length. Nevertheless our algorithm tolerates unreasonable parameter settings, because then it either simply rejects the corresponding alignment space transitions (though with some degradation in performance) and/or learns to avoid applying useless operations through its memory module, as described below.

### High level sampling strategies

Having specified various operations on the alignment space, we now need to specify when and how often to apply them, as well as how to escape from local traps and thus to most rapidly converge on an optimum or nearly optimum alignment.

#### Providing the sampler with a memory

Since some of the alignment operations are computationally expensive, it would be helpful to avoid applying them over and over again when this proves to be unfruitful. For example, if the sampler has already converged on the correct number of motifs, applying the AddBlock operation may be a waste of time. On the other hand, we don't want to eliminate any operation entirely, as at some point it may be useful. To do this we define both short-term and long-term sampling memories. The short-term memory allows a rapid response to sudden changes while the long-term memory adds stability so that the sampler does not over respond to short term trends. Details are given in Methods.

#### Simulated annealing with a thermostat

Let the target alignment distribution be denoted generically as *π *(**X**). As the sampler converges on near optimum alignments, typically it has difficulty 'dropping' into the global optimum of *π *(**X**) because the chance of selecting the highest probability alignment is still very small due to the sheer number of near optimum alignments. This is true for the same reason that the most likely outcome of obtaining exactly 5,000 heads and 5,000 tails in 10,000 flips of a fair coin is extremely unlikely.

A standard way around this problem is to take power of *π *(**X**) to some exponent, renormalizing it and using the "powered-up" distribution, denoted as *π*_*T*_(**X**) ∝ *π*^1/*T *^(**X**) with the "temperature" parameter varying from a very large value to near-zero, for sampling. This procedure is a key component of simulated annealing [29], which has the same effect on sampling as lowering the temperature has on annealing of single stranded DNA into double stranded DNA in solution. By 'cooling' the system (i.e., letting *T *→ 0), we raise the probability of high-density points and lower the probability of low-density points, so as to allow the best alignment to win out over alignments that are nearly as good. If the temperature is lower too abruptly, however, the sampler may get trapped in a sub-optimum alignment, so that the annealing strategy needs to be devised carefully.

We have built a 'thermostat' into the sampler that keeps track of variations in the (*T *= 1) probability densities of the sampled alignments. If the variance of log *π *(**X**) in a given number *K *of consecutive iterations at a given temperature is below a certain threshold (so that the posterior probabilities barely change), the sampler may be stuck in a (presumably local) optimum, and the thermostat raises the temperature a bit. On the other hand, if the log *π *(**X**) are varying wildly and, in particular, if they are greatly diverging from the best (i.e., highest probability) alignment found thus far, then the sampler may be wandering away from near optimum alignments and the thermostat lowers the temperature. This approach thus attempts to keep the sampler just above its 'glass transition temperature' [31], designated *T*_*g*_. Details are given in Methods.

Since there are no absolute criteria for determining whether the sampler has actually found the optimum alignment, it is necessary to devise heuristics for terminating the computation. We retain the same criterion used in earlier Gibbs samplers, such that if the alignment fails to improve after a specified number of sampling cycles, then the program stops and returns the best alignment found. Since picking the right number of cycles depends heavily on the number and nature of the input sequences (as well as the user's patience), the user can modify this parameter. As an alternative strategy, two or more programs may also be run in parallel until they both converge on the same alignment.

#### Progressive refinement strategy

When painting a picture, it is helpful to first draw a rough sketch so that details will end up in the right place relative to each other. Similarly the sampler uses the following progressive refinement strategy to avoid being too "shortsighted."

There are five stages to this strategy. In the first stage, the sampler applies the Align operation, which aligns the sequences against contiguous ungapped blocks; it also applies compound ungapped motif operations. The initial numbers of block motifs and columns in each block are sampled from binomial distributions with means between roughly 5~10 blocks and 10~30 columns each, respectively. In the second stage, which is introduced after the sampler begins to converge on a local optimum under the ungapped block-motif model, elementary and compound column operations are introduced, which allow these ungapped blocks to 'fragment', thereby permitting nonconserved columns to be ignored by the alignment model (mathematical details are found in [6]). Recombination operations are also applied during and after this stage. In the third stage, the GapAlign operation based on a simple gapped sampling procedure [14] with very conservative gap penalties is introduced, which allows the sampler to add short gaps within motif blocks and to delete part or all of a block. In the fourth stage, the number of blocks is fixed (although other operations are retained) and recombination and simulated annealing procedures are used to help guide the sampler into a (hopefully) global optimum. These first four stages are implemented in the program GISMO (see below). A fifth stage, which is implemented in the program GARMA (see below), recombines a set of alignments independently found by GISMO and optimizes the recombinants using a GapAlign procedure based on the HMM model described above. (Here we apply another annealing strategy, termed prior annealing, where early on low HMM gap penalty priors are used to introduce gaps more liberally, and later high HMM gap penalty priors are used to eliminate less convincing gaps.) GapAlign sampling is performed by Viterbi alignment of the sequence against the HMM where the HMM emission and transition probably parameters are sampled from the posterior distribution. Afterwards the resultant alignment is either rejected or accepted based on our new scoring function *g*(**A**, Λ).

#### Manual application of alignment operations

Despite attempts to codify and fully automate optimization of a multiple sequence alignment, the algorithm may still create an alignment model that lacks certain properties observed to be biologically important for a particular class of proteins. Take the situation, for example, where a motif, which occurs as a single block in most of the proteins, is split in two by a sizable insertion in other proteins and where the sampler, due to the *a priori *parameter settings chosen before the analysis, fails to split this motif into two blocks. In this case, a biologically more meaningful alignment may be achieved by manually intervening to split this ungapped region (followed, ideally, by additional optimization via MCMC sampling perhaps using adjusted prior probabilities). To accommodate such tweaking, we thus allow manual application of various operations. We find that splitting and trimming of aligned blocks are particularly helpful in this regard. Such manually modified alignments then may be reintroduced into a population of similar alignments for recombination and selection via our genetic algorithm [5] followed by further optimization.

### Implementation and examples

The theoretical concepts and strategies just described were implemented in the programs GISMO (Gibbs-like sampling with multiple operations), GARMA (genetic algorithm for recombinant multiple alignment) and GAMBIT (gapped alignment with MCMC-based indel tempering). GARMA recombines the output alignments provided by GISMO and then applies simulated annealing strategies on the recombinants. GAMBIT performs on a single alignment the same optimization procedures that GARMA performs on recombinants. Manual application of alignment operations may be performed using another program, TweakAln. These programs along with sample alignments are available from the authors. Multiple alignment of thousands of sequences in this way may take substantial time (e.g., overnight on a 10-processor Linux cluster), but this is not critical because, once performed for a particular protein class, such an alignment can be updated readily by seeding the sampler with a previously optimized alignment. Here we apply these programs to several large protein classes within the context of CHAIN analysis, which is our primary reason for generating such alignments.

#### Application to CHAIN analysis

CHAIN analysis both decomposes into distinct categories and quantifies the sequence constraints associated with conserved patterns in a multiple alignment. This yields evolutionary clues regarding the underlying structural mechanisms presumably preserving these patterns. Aspects of these mechanisms can be inferred by comparing category-specific selective constraints with known structures of members of the protein class being investigated, as illustrated in three recent publications [3,32,33].

**Figure 4:**
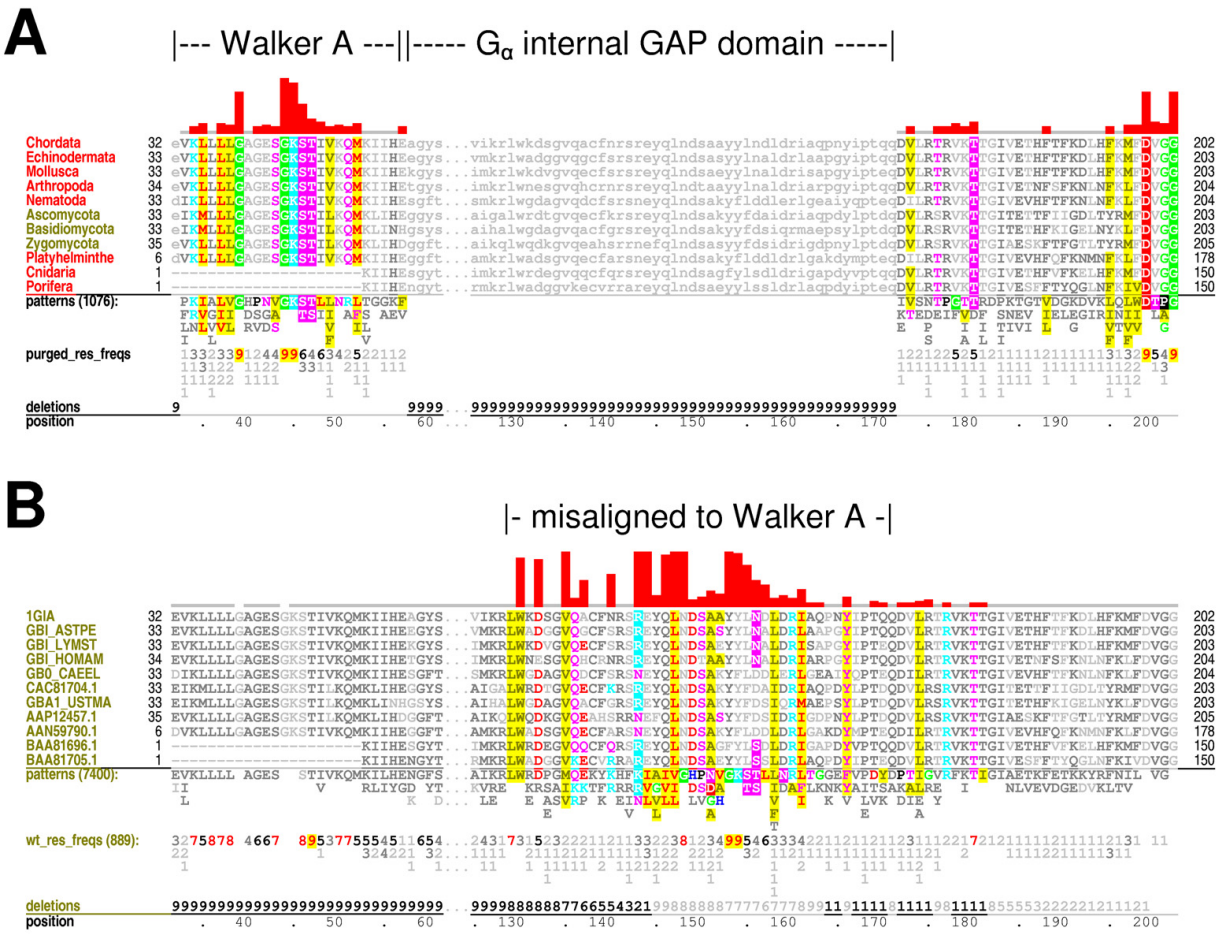
CHAIN analysis of P loop GTPase-specific constraints acting on the G_i*α *_subunit. The displayed sequences are representatives of the G_i*α *_family of G_*α *_proteins from distinct phyla. The foreground sequence set, which also includes P loop GTPases outside of the G_i*α *_family, are represented by the conserved residue patterns below the alignments. The number specified after the word 'pattern' gives the actual number of aligned sequences. **(A) **Motif-based contrast hierarchical alignment. Phyla are indicated in the leftmost column. Note that a purged foreground set [5] was used for the motif-based alignment in (A) and is thus smaller than the full set used by PSI-BLAST in (B). PSI-BLAST compensates for sequence redundancy by down weighting sequences [35] rather than by purging. The corresponding residue frequencies are given in integer tenths below the conserved patterns. For example, a '7' in integer tenths indicates that the corresponding residue directly above it occurs in 70–80% of the sequences. Deletion frequencies are similarly given in integer tenths (black; range 10–100%) or hundredths (gray; range 1–9%) as indicated. Histograms above the alignments display the strengths of the selective constraints acting at each position; aligned residues subject to the strongest constraints are highlighted for emphasis. For a complete description of CHAIN analysis alignments see [3]. **(B) **PSI-BLAST generated contrast hierarchical alignment. Sequence identifiers are indicated in the leftmost column. Note that, unlike the motif-based alignment in (A), PSI-BLAST misaligns the foreground set's Walker A region (represented by the patterns below the displayed alignment). In order to accentuate this alignment error, the background alignment in (B) consists of the foreground alignment in (A).

**Figure 5:**
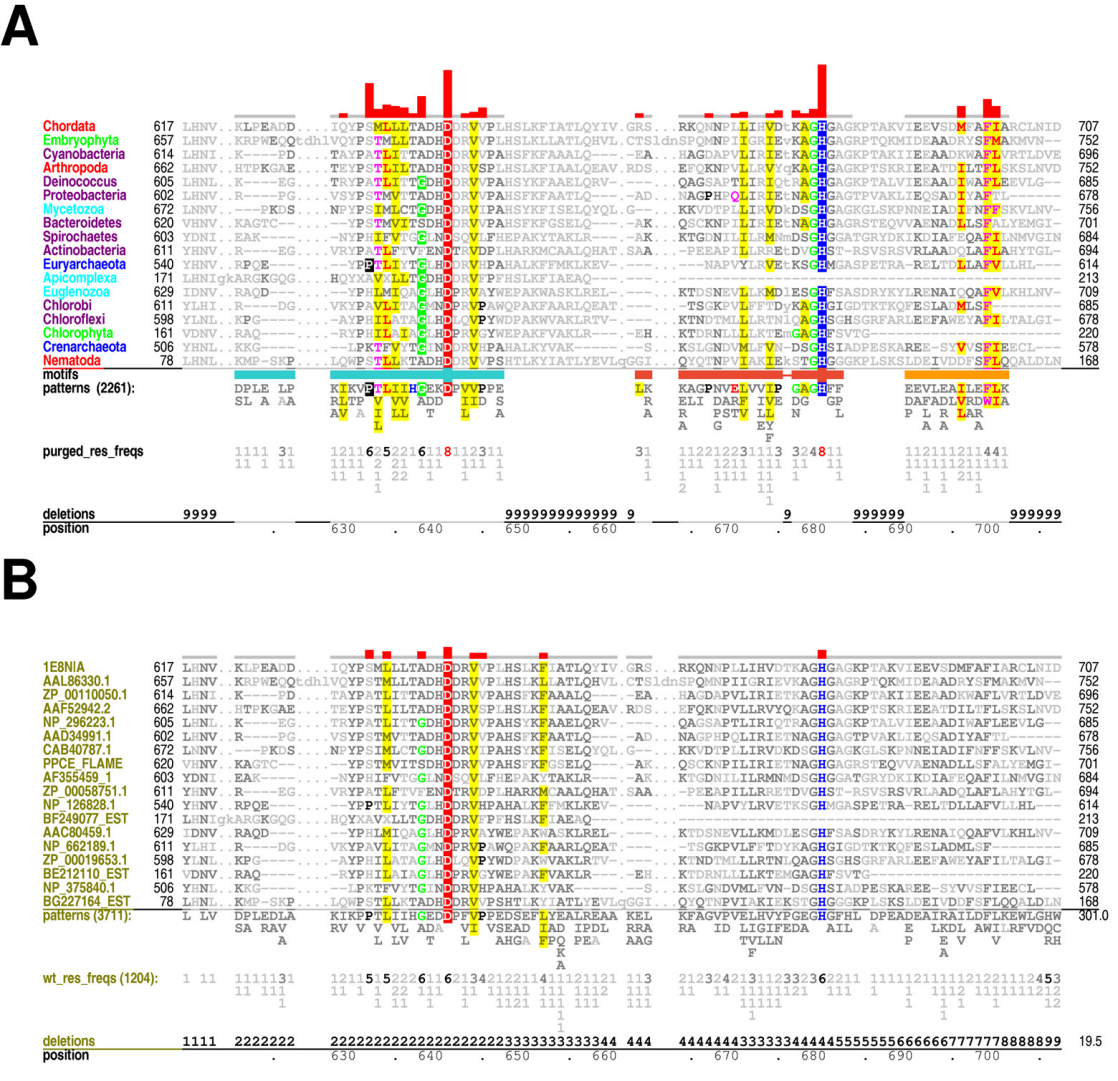
CHAIN analysis of *α*,*β*-hydrolase fold constraints acting on prolyl oligopeptidases. See legend to Fig. 4 for descriptions. **(A) **Motif-based contrast hierarchical alignment. The bars directly below the displayed sequences indicate motifs with the narrow line indicating a deletion relative to that motif and wide bars indicating catalytic residues. **(B) **PSI-BLAST generated contrast hierarchical alignment. The histogram heights for the catalytic aspartate and histidine in this alignment are shorter in this figure because, unlike the motif-based alignment in (A), the PSI-BLAST algorithm assigns relatively stronger constraints to the nucleophilic catalytic residue (not shown) that is much easier to align correctly.

**Figure 6:**
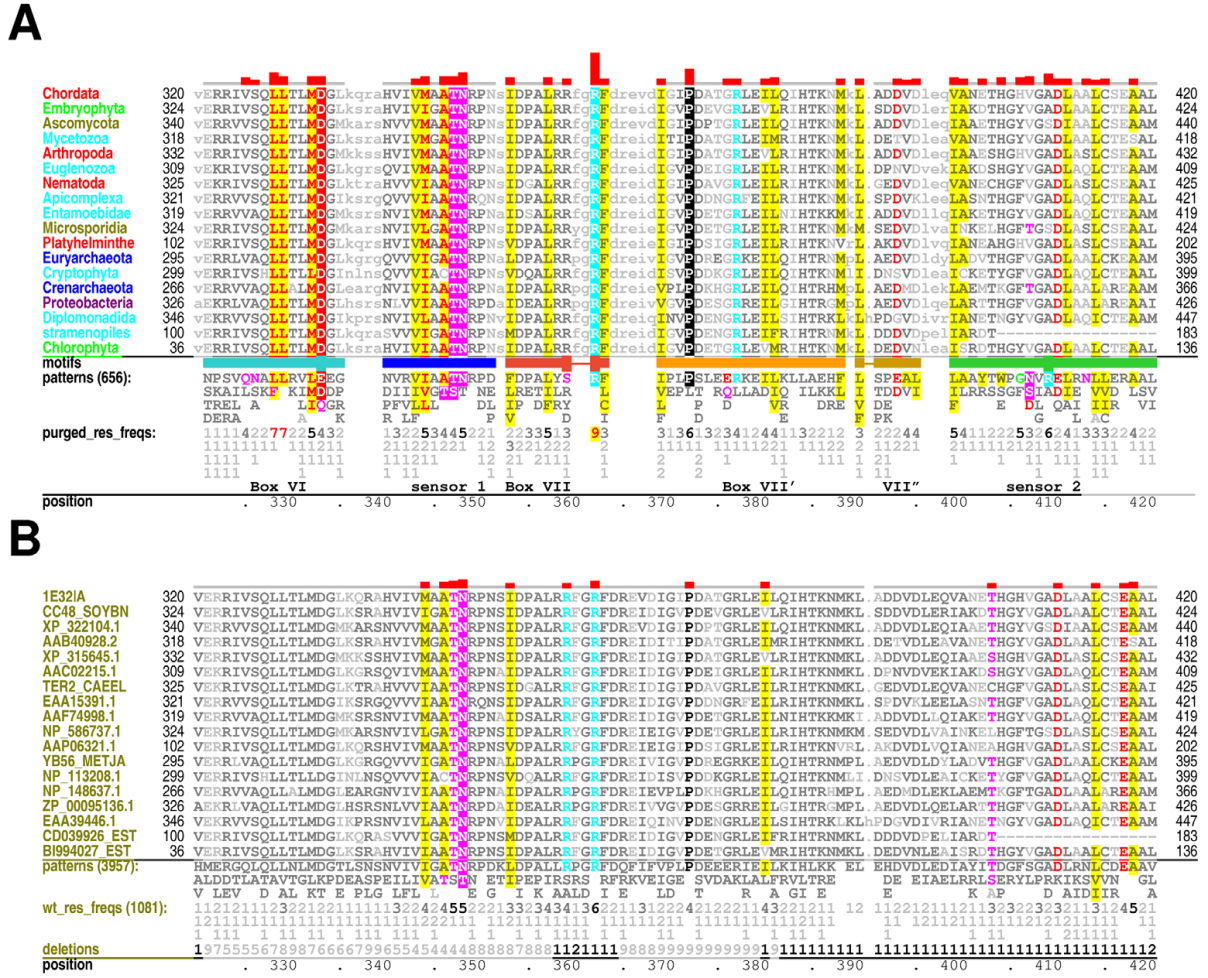
CHAIN analysis of the D1 AAA+ module of p97 ATPases. See legends to Figs 4 and 5 for descriptions and text for discussion. **(A) **Motif-based contrast hierarchical alignment. The bars directly below the aligned sequences indicate motif regions; wide bars indicate residues shown in Fig. 7 and discussed in the text. **(B) **PSI-BLAST generated contrast hierarchical alignment.

'Contrast hierarchical alignments', such as are shown in Figs 4,5,6, are the primary output from CHAIN analysis. In constructing such an alignment, three sets of related sequences are multiply aligned: (i) a 'displayed set', (ii) a 'foreground set', which is a superset of the displayed set, and (iii) a 'background set'. The displayed set corresponds to the aligned sequences of interest within the foreground set (i.e., only the alignment for these sequences is actually shown). The foreground set corresponds to the sequences whose selective constraints are being measured. These are not shown explicitly, but rather are merely represented by conserved patterns and residue frequencies shown below the displayed alignment (as in Fig. 4A). The original CHAIN analysis procedure uses a modified version of the PSI-BLAST algorithm to align these sequences. Here these PSI-BLAST alignments are compared with motif-based foreground alignments created using GISMO, GARMA, GAMBIT, and TweakAln.

CHAIN analysis measures selective constraints in terms of the difficulty of randomly drawing the amino acids observed at a particular position in the foreground alignment from the distribution at that position in the background alignment. In the examples here, unless specified otherwise, the overall frequency of amino acids generally observed in proteins serves as an implicit background set at each position. Foreground positions with compositions closely resembling the background presumably are subject to little or no selective constraints, while positions with compositions strikingly different from (i.e., that contrast with) the background are subject to strong constraints. In Figs 4,5,6 these constraints are displayed in the histograms above the alignments.

#### G_*α *_and P loop GTPases

We first examine in this way G protein *α *subunits. G proteins [17] are heterotrimers, consisting of an *α*, a *β *and a *γ *subunit, that mediate transduction of extracellular signals to the cellular interior. As do many members of the P loop GTPase class, the G_*α *_subunit functions as a binary switch that is turned on by binding GTP in response to the signal and thereby relays this information to downstream components of the pathway. This switch is turned off by hydrolysis of GTP to GDP, an event mediated by GTPase activating proteins (GAPs).

G_*α *_subunits are unique among such GTPase switches inasmuch as their GAP domain is contained within the G_*α *_polypeptide chain itself rather than existing as a distinct protein. This unique arrangement presents particular difficulties for CHAIN analysis because, during subsequent iterations, the PSI-BLAST algorithm tends to slightly overextend the alignment beyond G_*α*_'s region of homology to other P loop GTPases and into the C-terminal region of the GAP domain. As a result, the foreground patterns for the Walker A motif are mistakenly aligned against the C-terminal end of the GAP domain (Fig. 4B). By contrast, the Gibbs sampler avoids this misalignment problem because it can readily jump over the internal GAP domain (Fig. 4A). This thus illustrates how our motif-based approach avoids a serious problem encountered by PSI-BLAST.

#### *α*,*β*-hydrolase fold enzymes

Similar misalignment problems may be encountered between motif regions even when the aligned proteins lack large inserts. This is seen, for example, when aligning *α*,*β*-hydrolase fold proteins [18,19], which correspond to a large class of enzymes possessing a catalytic triad (typically consisting of a serine, an aspartate and a histidine) at their active sites. These three residues are involved in an electron transfer mechanism and thus are generally very highly conserved, despite the often very weak pairwise similarity between many members of this class. CHAIN analyses of prolyl oligopeptidases reveals that our motif-based alignment assigns very strong selective constraints to all three of these catalytic residues, the aspartate and histidine of which are shown in Fig. 5A. This is as expected, because conservation of one member of the catalytic triad is highly correlated with conservation of the other two, as the *α*,*β*-hydrolase electron transfer mechanism requires all three residues. In contrast, the PSI-BLAST alignment assigns a strong selective constraint to the catalytic serine (not shown in Fig. 5) but much weaker constraints to these other two catalytic residues (Fig. 5B). This is because the PSI-BLAST algorithm finds it much easier to correctly align the catalytic serine but, due to weak sequence similarity, often either misaligns or fails to extend the alignment into the C-terminal region of this domain. (The fraction of sequences that fail to align with this region is indicated near the bottom of Fig. 5B). Thus our motif-based approach again provides a better measure of the selective constraints acting on these residues.

#### P97 an AAA+ ATPase

**Figure 7:**
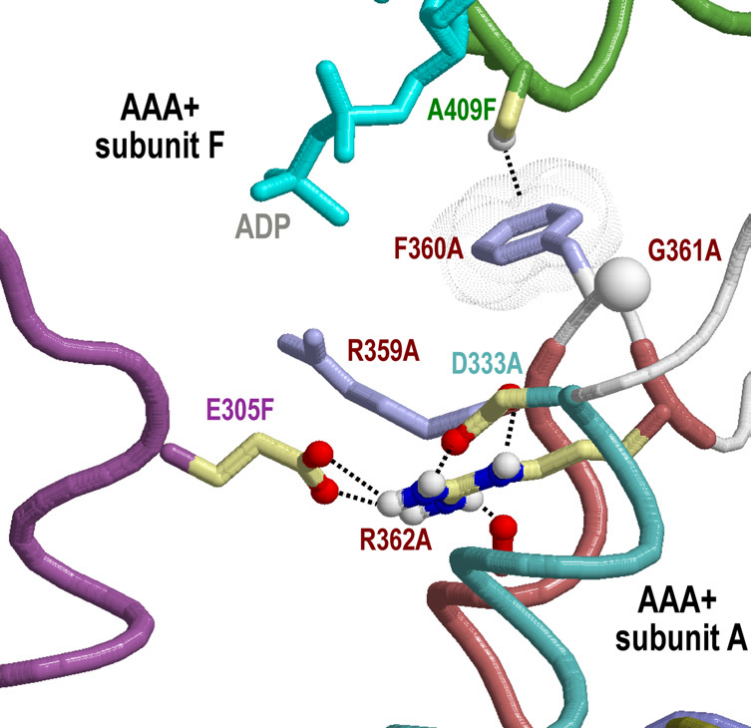
Structural location of the two-residue insert in Box VII of p97. The structure of the first domain (D1) of p97 from rat [34] is shown. The corresponding alignment is shown in Fig. 6.

Improved identification of a short insertion within a motif by our approach is illustrated through CHAIN analysis of p97, a transitional endoplasmic reticulum AAA+ ATPase (recently reviewed in [20]). AAA+ ATPases are a large and diverse class of chaperone and chaperone-like proteins [14,24,25]. They are characterized by the presence of one or more AAA+ modules, each of which consists of an *α*,*β*-fold domain, which it shares with other P loop NTPases, followed by a helical bundle domain. P97 contains two AAA+ modules, designated D1 and D2; our analysis was performed on the D1 module, whose structure is known [34]. These AAA+ modules often associate to form homohexameric complexes such that a prominently conserved arginine (R362A in Fig. 6 and 7) and a conserved acidic residue (D333 in Figs 6 and 7) in one module are positioned near a Walker B conserved acidic residue (E305 in Fig. 7) and a bound ATP-Mg^2+ ^in an adjacent AAA+ module.

When our motif-based approach was applied (with prior annealing) to AAA+ ATPases (Fig. 6A), it introduced within the Box VII motif of the p97 D1 module a two-residue insertion (most often a phe-gly; F360-G361 in Figs 6 and 7) immediately before a prominently conserved arginine (R362). By contrast, the PSI-BLAST alignment tends to misalign this region and, consequently, obscures both the two-residue insertion and the prominence of the conserved arginine (as indicated by the histogram height over this position; see Fig. 6B). The phenylalanine within this insert forms a CH-*π *interaction with an alanine (A409 in Figs 6 and 7) within the adjacent AAA+ module's three-helix bundle domain. Notably, an arginine often occurs at this alanine position in related AAA+ modules and is believed to sense bound ATP in the adjacent AAA+ module. (The region containing this arginine thus is termed the 'sensor II region'.) PSI-BLAST again does a poorer job aligning this sensor II arginine against A409 of p97 compared with our motif-based method. The improved motif-based alignment thus better reveals how the p97 AAA+ D1 module presumably utilizes an alternative configuration for sensing and responding to bound nucleotide relative to typical AAA+ modules (Fig. 7). In particular, two highly conserved p97 family-specific features – namely the phe-gly insertion, which is highly conserved in eukaryotes though replaced by a pro-gly in eubacteria and archaea, along with a third well conserved arginine directly preceding this insert (R359 in Figs 6 and 7) – are likely to perform an important role associated with p97's unique cellular function.

## Conclusions

With a view to improving alignments for CHAIN analysis, we have enhanced our earlier motif-based methods by developing (i) a HMM for insertions and deletions within motifs, (ii) an expanded algebraic system of operations on multiple alignments and (iii) various annealing and sampling strategies that facilitate rapid convergence on optimum or near optimum alignments. Furthermore, our approach, due to its rigorous statistical basis, fills a gap left by current multiple alignment methods inasmuch as it aligns only those characteristics of the input sequences that may be justified statistically. Thus it is useful for statistical analysis of conserved patterns in multiple alignments. Our statistical model likewise provides objective criteria for evaluating curated alignments, thereby guiding manual application of various operations. In the future, our MCMC sampling methods could be used to estimate alignment uncertainties, which will be useful for estimating background amino acid frequencies for CHAIN analysis. These approaches also serve as a starting point for further enhancements that integrate MCMC sampling, HMM and PSI-BLAST methods, which, based on our earlier analyses [16], seem likely to improve both alignment accuracy and search sensitivity.

When this motif-based approach was applied to CHAIN analysis of families belonging to large and diverse protein classes, we found numerous examples, three of which are described here, where this does a better job of revealing subtle, biologically important sequence features than does PSI-BLAST. This is in large part due to the ability of our statistical model and sampling strategies to find weakly conserved islands of homology within a sea of essentially nonconserved regions. While this motif based approach will not become the default method for CHAIN analysis – especially considering that PSI-BLAST alignments also may be optimized using these approaches – it, nevertheless, often more accurately aligns very distantly related sequences and thus can provide a better measure of selective constraints in this situation.

## Methods

### HMM architecture

We model gaps within motif blocks through the HMM shown in Fig. 2. The corresponding probability matrix for transitions between HMM states internal to the *i*th motif is:


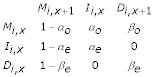


where 1 ≤ *i *≤ *m *and 1 ≤ *x *<*w*_*i *_and where M, I, and D denote match, insertion and deletion states, respectively. The probability matrix for transitions between motifs is:


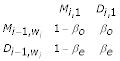


where 1 <*i *<*m *and where these transitions each emit a string of zero or more residues. Note that the contribution to the log-posterior probability of the lengths of these strings and of their emission probabilities (as well as those of M and I states) are specified by our ungapped statistical model [6], upon which this HMM is based and thus are unspecified by the HMM. Note also that the treatment we provide here easily can be generalized to cases where transitions I → D and D → I are allowed or where gap penalties are motif-specific.

### Statistical inference of indel penalties

For a given alignment ***A***, let *f*(***A***) be its log-posterior probability as in [6]. If we allow insertions and deletions within motifs, then each motif *i *within each sequence *S*_*k *_is associated with a "path" through the HMM indicating its alignment against motif model Θ_*i*_. Let the collection of these paths be Λ. Next, we denote the total number of transitions of type M → M, M → I, ..., by

*N*_*mm*_, *N*_*mi*_, *N*_*md*_, *N*_*im*_, *N*_*ii*_, *N*_*dm*_, *N*_*dd*_.

It then follows that the likelihood of the gap parameters is


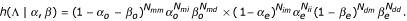


with independent prior distributions

(*α*_*o*_, *β*_*o*_, 1 - *α*_*o *_- *β*_*o*_) ~ Dirichlet(*a*_*o*_, *b*_*o*_, *n*_*m *_- *a*_*o *_- *b*_*o*_),

*α*_*e *_~ Beta(*a*_*e*_, *n*_*i *_- *a*_*e*_), and *β*_*e *_~ Beta(*b*_*e*_, *n*_*d *_- *b*_*e*_),

where *a*_*o*_, *b*_*o*_, *n*_*m*_, *a*_*e*_, *n*_*i*_, *b*_*e*_, *n*_*d *_are prior pseudo counts given by the user. The corresponding maximum likelihood estimates (MLEs) are


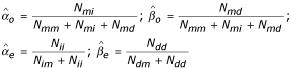


The joint posterior distribution for the alignment and gap parameters is

*g*(**A**, Λ, *α*, *β*) ∝ *P*(**S **| **A**, Λ) × *P*(**A**) Λ *h*(Λ | *α*, *β*) *P*(*α*, *β*),

where *P*(**S **| **A**, Λ) × *P*(**A**) is computed the same way as in the original block-motif model [6], and

*P*(*α*, *β*) = Dirichlet(*a*_*o*_, *b*_*o*_, *n*_*m *_- *a*_*o *_- *b*_*o*_) × Beta(*a*_*e*_, *n*_*i *_- *a*_*e) *_× Beta(*b*_*e*_, *n*_*d *_- *b*_*e*_).

Given the alignment Λ, we have the conditional posterior distribution


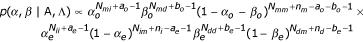


Sampling on this distribution can be performed by drawing the following random variables:


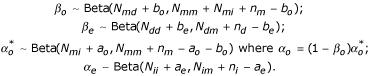


### Parameter collapsing

For computational efficiency, we integrate out the *α *and *β *to get


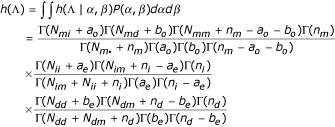


This gives rise to a new posterior *g*(**A**, Λ) with *h*(Λ) replacing *h*(Λ | *α*, *β*) *P*(*α*, *β*) in our previous formula [6] and frees us from having to fix or update the gap parameters. This also allows us to determine the optimum posterior gap penalties based on the sequence data.

### Prior specifications

Suppose that we expect to see one insertion in every *K*_1 _residues and one deletion in every *K*_2 _residues. Then we set


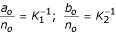


and set *n*_*o *_to reflect the strength of this conviction. We suggest using priors reflecting conservative gapping where, for example, *K*_1 _= *K*_2 _= 1000 and *n*_*o *_= *n*_*M *_*N*, where 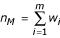 is the total number of match positions in all of the motifs and *N *is the total number of aligned sequences.

For gap extension prior probabilities, if one expects to see an average insertion length of *L*_1_, and deletion length of *L*_2_, then we let


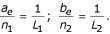


We set the prior pseudo counts *n*_1 _to be equal to the total number of expected insertions within motifs *n*_*M *_/ *K*_1_. Likewise, *n*_2 _is set equal to the expected number of deletions *n*_*M *_/ *K*_2_. In order to have different gap parameters for each motif, one need only keep specific counts of insertions and deletions for each motif, as the formula *h*(Λ) then applies to each motif individually, and we only need to multiply these *h*( ) functions together when computing the total 'penalty'.

### The sampler's memory

For long-term memory we monitor among the sampler's previous iterations the number of times *N*_*o *_(where typically, *N*_*o *_= 25) that a type "*o*" operation has been applied and the number of times *n*_*o *_that it was "successful" (i.e., resulted in an increase of the posterior probability). The same is done for short-term memory except that in this case we monitor the number of short-term successes *m*_*o *_over *M*_*o *_previous applications (where typically *M*_*o *_= 5). At the next iteration, we then assign a probability 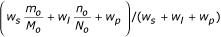 of applying this operation, where *w*_*s *_≥ 0 and *w*_*l *_≥ 0 are the weights given to the short and long-term memories, respectively, and where *w*_*p *_≥ 0 specifies the minimum frequency at which this operation is applied. Typically, we set *w*_*s *_= *w*_*l *_= 1 and 0.2 ≤ *w*_*p *_≤ 0.66, so that operations that previously proved to be unfruitful will only be performed about one-tenth to one-third as often as those that always yield improvements in the alignment.

### The sampler's thermostat

We define an intuitive sampling temperature *T*' = 300/*T *and, thus, *π*_*T*'_(**X**) ∝ *π*^300/*T *^(**X**). On this 'pseudo-degrees-Kelvin' scale sampling from the true distribution *π *(**X**) (i.e., 300°) corresponds to sampling at 'room temperature'. After a period of sampling at room temperature until 'convergence', which is defined by the sampler's failure to improve the MAP after a specified number of iterations, simulated annealing is initiated. During this stage, whenever the probability densities of the sampled alignments averaged over say 20 iterations fluctuate by more than some maximal value, say Δlog(*p) *≥ 50 nats, the temperature is lowered by 1–5°. If, on the other hand, the probability densities of the sampled alignments fluctuate on average less than some minimal value, say Δlog(*p) *≤ 5 nats, the temperature is raised by say 1°. (The precise parameters used are not critical and may depend somewhat on the input sequence set.) This period of thermostatic sampling is again applied until convergence.

## Authors' contributions

AFN developed the algorithmic strategies and early *ad hoc *approaches conceptually similar to the statistically rigorous procedures described in Methods, which were designed by JSL. AFN implemented the procedures and performed the sequence analyses. Both authors wrote and approved the final manuscript.
